# Tentacle: distributed quantification of genes in metagenomes

**DOI:** 10.1186/s13742-015-0078-1

**Published:** 2015-09-07

**Authors:** Fredrik Boulund, Anders Sjögren, Erik Kristiansson

**Affiliations:** Division of Statistics, Department of Mathematical Sciences, Chalmers University of Technology and University of Gothenburg, Gothenburg, Sweden

**Keywords:** Distributed computing, Master-worker, Next-generation sequencing, Metagenomics, Gene quantification, DNA sequence analysis, Read mapping, DNA sequencing

## Abstract

**Background:**

In metagenomics, microbial communities are sequenced at increasingly high resolution, generating datasets with billions of DNA fragments. Novel methods that can efficiently process the growing volumes of sequence data are necessary for the accurate analysis and interpretation of existing and upcoming metagenomes.

**Findings:**

Here we present Tentacle, which is a novel framework that uses distributed computational resources for gene quantification in metagenomes. Tentacle is implemented using a dynamic master-worker approach in which DNA fragments are streamed via a network and processed in parallel on worker nodes. Tentacle is modular, extensible, and comes with support for six commonly used sequence aligners. It is easy to adapt Tentacle to different applications in metagenomics and easy to integrate into existing workflows.

**Conclusions:**

Evaluations show that Tentacle scales very well with increasing computing resources. We illustrate the versatility of Tentacle on three different use cases. Tentacle is written for Linux in Python 2.7 and is published as open source under the GNU General Public License (v3). Documentation, tutorials, installation instructions, and the source code are freely available online at: http://bioinformatics.math.chalmers.se/tentacle.

**Electronic supplementary material:**

The online version of this article (doi:10.1186/s13742-015-0078-1) contains supplementary material, which is available to authorized users.

## Findings

### Introduction

The development of next generation sequencing technology has resulted in unprecedented volumes of data being generated by the research community [1]. Central repositories for nucleotide sequence data, such as The European Nucleotide Archive, have observed a doubling in size every 10 months, a rate of increase that is predicted to hold for the coming years [2]. The increase in the output of DNA sequencing technologies is outpacing Moore’s Law, rendering single-computer systems increasingly impractical for the necessary data processing. The development of methods and algorithms that efficiently use distributed computer systems is vital to the analysis of modern sequence datasets within reasonable time frames [3].

In metagenomics, complex mixtures of microorganisms are studied by sequencing random fragments of their genomes [4]. A single sample from, for example, soil, sediment, or marine water can contain millions of cells from tens of thousands of species, making the total genetic content massive [5]. Consequently, metagenomic studies generate sequence data on the order of terabases (10^12^ nucleotides) [6, 7], and these numbers are predicted to continue to increase. For example, the ongoing Earth Microbiome Project aims to generate petabases of metagenomic data (10^15^ nucleotides) over the coming years [5, 8, 9].

The quantification of genes is an essential step to understand, interpret, and compare metagenomes. This process consists of three main steps: i) quality assessment and filtering of the input data; ii) alignment of the reads to a reference database; and iii) estimation of the gene abundances. The reference database, typically containing a gene catalog, a collection of contigs, or genomes, is often incomplete because most microorganisms encountered in metagenomes lack a sequenced reference genome. For example, although there are approximately 56,000 sequencing projects listed in the Genomes OnLine Database (GOLD) [10], this only reflects a tiny part of the total microbial biodiversity, which is estimated to have more than 10 million species [11]. Therefore, highly sensitive alignment algorithms are necessary to match as many fragments as possible to related sequences in the reference database. Although many algorithms have been developed for this purpose [12], high sensitivity and specificity increases the computational costs and many existing single-computer methods are not practically applicable to the size of modern metagenomes [13].

Distributed computing resources, such as clusters and clouds, can consist of up to tens of thousands of interconnected computers. Their combined power has the potential to enable processing of large volumes of DNA sequence data within practical time frames. However, distributed computer systems require methods that can efficiently disseminate the data and distribute the computational tasks. Consequently, a wide range of methods have been developed for distributed sequence alignment. For example, several BLAST-based approaches that distribute the well-known algorithm [14] have been developed (such as BeoBLAST [15], Squid [16], G-BLAST [17], mpiBLAST [18], Soap-HT-BLAST [19], W.ND-BLAST [20], and CloVR [21]). DistMap is another method for distributed sequence alignment [22], which is cloud-based and can use multiple sequence aligners. Other cloud-based methods include CloudBurst [23], CloudAligner [24], and STORMSeq [25], which implements complete analysis pipelines for the discovery of single nucleotide polymorphisms. In addition, QIIME [26] offers support for distributed analysis and visualization of marker gene survey data (such as 16S, 18S, and ITS amplicon data) together with limited support for shotgun metagenomics. However, most of these methods are focused on sequence alignment and many lack the functionality for distributed sequence quality assessment and gene abundance estimation. Furthermore, few of these methods are designed for processing terabase-sized shotgun sequencing datasets, making them unsuitable or difficult to apply to the analysis of modern metagenomes.

In this paper we present the Tentacle framework for distributed gene quantification in metagenomes. Tentacle uses a dynamic master-worker approach where sequence data is streamed over the network and processed in parallel on worker nodes while keeping the number of time-consuming disk operations to a minimum. Tentacle supports six commonly used read mappers, which makes it applicable to all forms of gene quantification tasks.

### Implementation

**Fig. 1:**
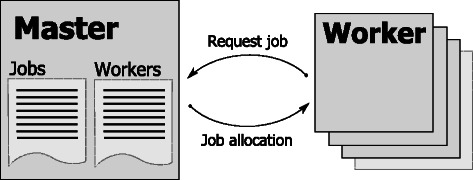
Master-Worker. Tentacle uses a dynamic master-worker scheme to distribute work between computer cluster nodes. The master maintains a list of jobs to be run and a list of available worker nodes. Worker nodes can dynamically come online at any time and register with the master process to start receiving jobs. When the workers finish a job, they report back to the master for another job. This continues until the master’s job queue is depleted. The master process can request that worker nodes rerun jobs that fail

Tentacle stratifies metagenomes into jobs that are distributed using a dynamic master-worker scheme (Fig. 1). The master is initialized with a list of jobs, each containing file paths to three items in the distributed file system, as follows: i) a set of reads; ii) a reference database; and, iii) a file with annotations of the data in the reference database. Worker nodes dynamically register to the master process as they become available and they are then provided with a job. The dynamic master-worker scheme allows worker nodes to be heterogeneous (for example, with regards to memory and CPU configuration) and does not require them to be allocated simultaneously. After a job is finished, the worker notifies the master, which in turn provides a new job. This continues until the list of jobs is exhausted. The master maintains the list of currently running jobs and can request worker nodes to rerun jobs that encounter errors. The status of running jobs can be interactively queried and listed using a supplied, easy-to-use command-line tool. Communication between master and worker processes is implemented using the high performance messaging library ZeroMQ [27].

The workflow has been designed to accept common standard sequence formats. Reads can be supplied in the ubiquitous FASTA or FASTQ formats, and will be automatically converted in the Tentacle pipeline to the correct format for the selected alignment program. The reference database is supplied in FASTA format, and in some cases formats specific to some mappers. The reference database can contain anything that the chosen mapper will align against (for example, a selection of reference genomes, single genes, or assembled contigs, as nucleotide or amino acid sequences depending on mapper). The annotation of the reference sequences is supplied as a simple tab-separated format, akin to a simplified version of the General Feature Format (GFF).

**Fig. 2:**
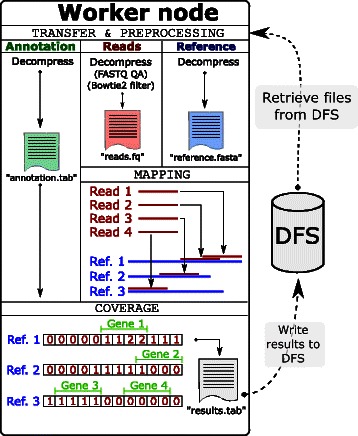
Overview of the data analysis pipeline executed on each worker node. The workers perform transfer of compressed data files from the distributed file system (DFS), pre-processing (such as FASTQ quality filtering/trimming or removal of human sequences using Bowtie 2), read mapping, and counts/coverage calculation. Note that ambiguously mapped reads are not depicted in this figure; the user can choose whether to keep all of the mapped reads or keep only the best match. Worker nodes fetch their data independently of the master process, thus minimizing the risk for data transfer bottlenecks. It is possible to instruct Tentacle to retrieve mapping results from worker nodes after mapping is completed. Counts/coverage calculations can be disabled, which when combined with retrieval of mapping results effectively transforms Tentacle into a parallel mapping framework

The bioinformatics data processing pipeline executed on each node consists of three overall steps: i) data transfer and pre-processing; ii) mapping of reads to the reference; and iii) estimation of coverage (Fig. 2). The first step performs data transfer and pre-processing in a single unified pipeline. Data is transferred directly from the distributed file system and is decompressed into a continuous in-memory stream, minimizing the number of inefficient disk read and write operations. Sequence reads can be quality assessed and filtered using FASTX [28]. As an additional pre-processing step, Bowtie 2 [29] can be used to identify and remove sequence reads matching (for example, the human genome), which is useful, for example, when working with metagenomic data from human microbiomes.

In the read mapping step, the reads are aligned to the reference database and the mapping output is written to the local scratch disk of the nodes. The last step combines the mapping output with the reference annotation to compute coverage across the annotated regions of the reference sequences, before writing the final quantification results back to the distributed file system. It is highly important that the worker nodes are independent of the master process for everything but receiving the job description (i.e., paths to job-relevant files) to reduce the risk of the master process becoming a bottleneck. The implementation is, therefore, designed so that the master process is never involved in the transfer of data or results. The data and results are instead read from and written to the distributed file system directly by the workers.

In the quantification step, Tentacle can compute both counts and coverage for annotated regions in the reference database. Counts are the number of reads that align to each annotated region (based on a user-definable overlap criteria). Users can select one of two options for handling ambiguously mapped reads: i) only use the ’best’ hit (defined as the first listed hit in the mapper output); or, ii) use all hits that meet the user controlled matching criteria. Tentacle has implemented coverage calculations in the following way. Each sequence in the reference database is represented by an array of integers. For each mapped read the integers at the start and end positions in the corresponding reference sequence array are incremented and decremented by 1, respectively. After all of the +1 and −1 contributions to the affected start and stop positions in the reference are handled, the total coverage across each reference sequence is calculated as the cumulative sum over the corresponding array. The operations are serially performed for each read and reference sequence. The calculation of coverage has a time complexity of $\mathcal {O}(n + N)$, where *n* is the total number of bases in the reference database and *N* is the number of mapped reads. This is substantially faster than the naïve approach that increments the coverage for each mapped base covered by a read, yielding a time complexity of $\mathcal {O}(n + N \cdot M)$, where *M* is the maximum number of bases per read. The output format for counts and coverage information across reference sequences is a simple tab separated file with fields for the reference sequence, annotation start/stop coordinates, counts, median coverage, mean coverage, and coverage standard deviation (see Additional file 1 for a detailed example). The user can choose to disable counts or coverage calculations independently of each other (both can be skipped, in effect reducing Tentacle to a framework for performing parallel read mapping using any of the supported aligners).

Because Tentacle wraps many third-party tools and runs a large number of processes simultaneously, it logs all events extensively. By default, Tentacle writes log files for each sample it processes and a processing log is produced by the corresponding worker. This is important for scientific reproducibility and traceability. Run settings are stored in a separate master log file, along with information about which worker node was assigned what job as well as other orchestration details. In addition to the master log file, additional processing logs are produced by each worker node as they handle jobs. There are two levels of log output in Tentacle. The default logging level is to print/write informational messages only (i.e., when important steps of the workflow took place). The second option is to print a verbose output with summary statistics before and after many steps in the workflow, which is useful for troubleshooting or to find and investigate issues with an analysis. If the user desires, the log output can also be written to the attached terminal that started Tentacle’s master process.

Tentacle can use several alignment algorithms. Currently, it supports six commonly used mappers: BLAST [14], pBLAT [30, 31], Bowtie 2 [29], GEM [32], RazerS3 [33], and USEARCH [34]. The mapping parameters of the mappers can be adjusted via Tentacle’s command line interface. The default settings for each mapper used in Tentacle are available as an additional file Additional file 2, and they are also listed in Tentacle’s online documentation [35]. The cluster scheduling software Slurm [36] is used to launch worker nodes. The framework can also be run locally on a single computer using multiple cores for parallelization (such as for testing or for smaller datasets). Tentacle is modular and can be easily extended (for details on how to modify or contribute to Tentacle, please refer to the online documentation [35]). Installation of the Tentacle Python package is easy and can be performed in a single command using Python’s package manager pip (installation instructions, along with tutorials and example data, are available on the project’s home page [35]).

### Results

#### Scaling

**Fig. 3:**
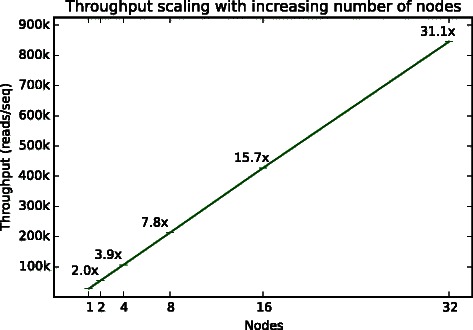
Scaling. This graph shows the average throughput of three identical runs per point. The axes show throughput (reads per second) versus number of worker nodes. The numbers at each point describe the increase in throughput compared with running on a single node. Standard errors for each point in the graph: 0.67,2.72,3.39,1.49,0.99,1.06 (standard error bars are barely visible in the figure). Note how Tentacle displays a near perfect scaling with increasing computing resources. The measurements were performed with replicates of a metagenomic sample from [6]

We evaluated the performance of the Tentacle framework by measuring the speedup achieved when increasing the number of engaged worker nodes. The evaluations were performed on a cluster comprising 379 nodes, each with 16 CPUs and at least 32 GiB of RAM. The nodes were connected to a distributed file system (DFS) via gigabit Ethernet. The evaluation data consisted of multiple copies of a single read file from the Meta-HIT study [6]. The read data in this study was produced using the Illumina Genome Analyser (GA). The reads in the samples used in this example were on average 45 base pairs long. The reads were mapped to the corresponding assembled contigs [6]. The number of reads in the sample was 11,706,305 and the number of contigs in the reference was 14,301. The pBLAT mapper was used to map the reads (options: -threads=16 -minIdentity=90 -out=blast8). Figure 3 displays how the throughput in reads per second scales with the number of nodes. With this specific combination of mapper, computer cluster, concurrent network usage, etc., Tentacle provided a throughput of more than 800,000 reads per second (about 2.9 billion reads per hour) using 32 worker nodes. The complete details of the evaluation are available in an IPython [37] notebook [38].

We also performed a comparison between the distributed BLAST search functionality of CloVR [21] and Tentacle. The evaluation was performed using 16 workers. CloVR was run in Oracle VirtualBox using the publicly available CloVR virtual machine image. Tentacle was run on the same physical machine in a native Linux system. The sample used in the evaluation contained 12 million metagenomic read fragments, which were mapped to the corresponding assembled contigs. The complete run time of the CloVR BLAST search pipeline was 15 hours and 9 minutes, whereas Tentacle finished analyzing the same data in 5 hours and 7 minutes. Thus, Tentacle was 3.0 times faster while still performing coverage calculations in addition to the mapping. When Tentacle was run on the same data using pBLAT, which is a more appropriate mapping method for the task, the work was finished in 6 minutes and 43 seconds, corresponding to a 135 times speedup (for complete details see Additional file 3).

#### Three use cases

To highlight the versatility of Tentacle, three different use cases with three different mappers were evaluated. The use cases are listed in Table 1 and they represent the following three scenarios: 1) mapping metagenomic reads to assembled and annotated contigs from the same metagenome; 2) mapping metagenomic reads to a large database of reference genes; and 3) mapping metagenomic reads to a database of amino acid sequences.

**Table 1:** Tentacle applied to three use cases. All use cases used the same read data consisting of 1,238,598,682 reads with a total size of 407 GiB in compressed FASTQ (2,213 GiB uncompressed) [6]. The examples were run on 30 nodes, with the cluster system login node hosting the master process. The following options were used, pBLAT: -threads=16 -minIdentity=90 -out=blast8; GEM: -T 16 -m 0.04 -e 0.04 –min-matched-bases 0.80 –granularity 2500000; USEARCH: -usearch_local -query_cov 1.0 -id 0.9 -blast6out

Use case	1	2	3
	Reads mapped to their contigs	Reads mapped to large DB	Reads mapped to peptide DB
Mapper	pBLAT	GEM	USEARCH
Type of reference	Per sample contigs (nucleotide) [6]	BGI Refseq geneset (nucleotide) [6]	Resqu; antibiotic resistance gene
			database (peptide) [53]
Reference size (bytes)	approx. 160 MiB per sample	3.0 GiB	1 MiB
Reference size (sequences)	6,589,348	3,305,138	3,019
Runtime (core hours)	720	3,072	296
Runtime (wall-clock)	1h 30m	6h 24m	0h 37m

Use case 1 corresponds to quantification of genes annotated in a metagenome. This requires read mapping with relatively low sensitivity because the reads can be expected to match with high similarity to the contigs created from the same reads. Use case 2 corresponds to a case when the gene abundances of a set of specific genes are compared between metagenomes. This requires sensitive mapping because the reads do not necessarily have high similarity with the genes in the reference database. Use case 3 corresponds to a similar use case as use case 2 but requires a translated search (i.e., like BLASTX [14]) because the references are protein sequences.

**Fig. 4:**
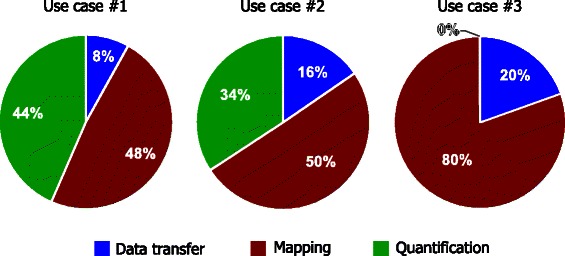
Relative times for subtasks in three use cases. Relative times are listed for: i) transfer and decompression and pre-processing of data files (reads, references, annotations); ii) mapping of reads; and, iii) computing coverage/counts. All of the examples were run with 512 samples of metagenomic data [6] on C3SE cluster Glenn using 30 nodes. Use case 1 mapped metagenomic reads to assembled contigs using pBLAT. Use case 2 mapped metagenomic reads to a large reference (3.0 GiB) database of genes using GEM. Use case 3 mapped metagenomic reads to a small gene database (1.0 MiB) of amino acid sequences. The label ‘data transfer’ includes time for transfer and on-the-fly decompression of reads, references, and annotations

Figure 4 displays how the workload on the worker nodes differs between the three use cases. For use case 1, the time to transfer and decompress data is small in relation to the time to map the reads and compute counts for each annotated region in the contigs. In use case 2, the time to transfer, decompress data, and map the reads, all require more time caused by the large reference database. Use case 3 highlights how mapping reads to a reference database containing amino acid sequences spends most of the time on mapping the reads (which includes translating the reads into all six reading frames). The time to compute quantification results for the third use case is negligible.

The total run times required to perform quantification in the three use cases are listed in Table 1. Use case 1 required a wall-clock time of 1 hour and 30 minutes on 30 nodes (a total of approximately 720 core-hours). Similarly, use case 2 required 6 hours and 24 minutes (30 nodes; 3072 core-hours) and use case 3 required 37 minutes using USEARCH with translated search (30 nodes; 296 core-hours). The time to run the same analysis on a powerful single computer would be approximately 30 times longer; that is, more than two days, eight weeks, and approximately 18 hours, for the three use cases, respectively.

#### Coverage and quantification accuracy

We performed a validation study to verify that Tentacle performs quantification of annotated regions as expected. Three samples from the Meta-HIT publication [6] were chosen at random. For each sample, 10 % of the corresponding contigs longer than 2500 nucleotides were randomly selected. The samples were then spiked with artificial reads (of the same length as the reads in the original sample) that were created by randomly fragmenting the selected contigs at 1 ×, 10 ×, 100 ×, and 1,000 × coverage. Tentacle was then run on the original and spiked samples, and the coverage of the selected contigs was estimated. The estimated median coverages were within 0.81 %, 0.98 %, 1.9 %, and 2.9 % of the expected theoretical coverages, for the 1 ×, 10 ×, 100 ×, and 1,000 × levels, respectively. Standard errors were 1.05×10^−4^, 2.01×10^−4^, 1.27×10^−3^, 2.84×10^−2^, respectively. A detailed description of the evaluation with explanations of the input data, computations, plots, and results is available in Additional file 4.

### Discussion

Tentacle is a novel framework for distributed quantification of genes in metagenomes. It was designed with flexibility in mind and can easily be integrated into existing workflows. The framework has been optimized to exhibit low overhead and minimal memory requirements by streaming data with few intermediate input/output operations. Therefore Tentacle is well-suited for rapid processing of very large volumes of metagenomic data. Our performance evaluation demonstrated that the method exhibits near perfect scaling with the number of used computer nodes.

The proposed framework applies a dynamic master-worker strategy (DMW) to estimate gene abundances in metagenomes. In contrast to the more traditional Single Program, Multiple Data (SPMD) approach [39] used with, for example, MPI [40], where tasks are split between a fixed number of nodes, DMW can dynamically add and remove workers as the availability of nodes in the cluster varies in real-time. The DMW approach can also use heterogeneous workers with regards to CPU and memory configuration without any special configuration. Additionally, the master can also monitor the status of the individual workers and disconnect nodes that encounters errors. These properties make DMW preferable for distributing large data over a high number of nodes (such as clouds) or on computer systems consisting of less reliable commodity hardware [41, 42]. MapReduce is an alternative distribution strategy to SPMD and DMW [43], which is implemented in the Hadoop framework [44] that is used by several other distributed mapping applications (for example [22–24]). Similarly to DMW, MapReduce can use dynamically changing resources and is robust against node failures. However, MapReduce in its general form cannot re-use data between jobs (for example, it cannot use the same reference database). It is perfectly possible to implement this in Tentacle by virtue of the DMW strategy. However, this implementation has not been a priority because it has not yet been required. This functionality is currently planned for future updates. Our method is light-weight with few dependencies and it can easily be deployed in most situations on most computer configurations with no or relatively minor modifications. The scaling results displayed in Fig. 3 also underline that our implementation of the DMW strategy provides excellent scaling characteristics, which makes it highly suitable for distributed analysis of large volumes of DNA sequence data.

The comparison with CloVR [21] highlights the efficiency of Tentacle’s DMW implementation. CloVR is distributed as a virtual machine image that contains a complete Ubuntu-based Linux system bundled with facilities and software for managing and running such pipelines. The administration of pipelines in CloVR is handled via a web browser interface. The virtual machine contains tools for running a Sun Grid Engine, which can be deployed to run distributed computing processes in a wide variety of computing environments. Our results show that Tentacle completed in a third of the total run time of CloVR while performing more work (both mapping and coverage calculations). As the results in Additional file 3 show, Tentacle is 135 times faster than CloVR when using an algorithm more suited for the mapping problem (pBLAT [31]). This example highlights that Tentacle is well suited for analyzing very large metagenomes.

The performance evaluation shows that the computational effort of processing typical metagenomic samples is dominated by mapping and quantification. These two processes are computationally intensive and the overhead required to distribute the data to the worker nodes remains a minor part of the total time. In use case 1, we see that mapping and quantification consume approximately the same amount of time, which is likely to be a result of the high similarity between reads and the reference contigs. The high similarity leads to many reads that map to the reference sequences, thus making the quantification (which must process the coordinates of all mapped reads) almost equally as time consuming as the mapping process. In use case 2, the time to map the reads is much longer than the time to perform the quantification. Given that use case 2 compares the reads to a database of gene sequences, fewer reads are expected to align to the references than in the previous use case. Despite the database being substantially larger than in use case 1, the time for quantification remains shorter than the mapping time, which is partially because of this process. In use case 3, the time for quantification is negligible compared to the time for USEARCH to perform translation and mapping of all of the reads. In all of the examples, the time for transfer and decompression of data consumes a minor portion of the total time (8–20 %).

The rapidly increasing amounts of information in molecular databases, including the many ongoing genome and metagenome sequencing projects, will result in the growth of reference databases. This will, in turn, result in an increase in time for data transfer and read mapping. The impact on mapping performance is, however, dependent on the algorithm and can, therefore, differ substantially depending on the method used [45, 46]. The size of the reference database can thus become an important parameter in the selection of a suitable mapper to use with Tentacle. It should, however, be noted that, regardless of the choice of read mapper used, the scaling properties of the Tentacle framework will remain consistent. Furthermore, if the reference database becomes so large that the mapping can no longer be accommodated entirely on a single worker node, then the versatility of the DMW architecture and the modularity of Tentacle makes it possible to circumvent such problems. For example, the data transfer logic can be modified to use more intelligent data transfer strategies that cache the data between consecutive runs. Furthermore, high-performance on-the-fly compression and decompression of the input metagenomic data and the reference databases can be used to reduce the data transfer time. Indeed, highly optimized compression algorithms have recently been developed for sequence data [47], which (in contrast to the ubiquitous gzip) could be used to achieve both an increased compression rate and a higher IO throughput in distributed frameworks such as Tentacle.

When discussing optimization by parallelization, it is important to keep the impact of Amdahl’s law [48] in mind. Amdahl’s law states that the maximum achievable speedup using *N* processors is $S(N) = \frac {1}{(1-P)+\frac {P}{N}}$, where *P* is the proportion of the program that can be parallelized. This relates to Tentacle in two ways: the first is that using worker nodes with multicore processing capabilities can help reduce the run time for individual subcomponents in the workflow if they are parallelized; and the second is that the data distribution problem that Tentacle sets out to solve is essentially perfectly parallel. Perfectly parallel problems have the property that the *P* component of Amdahl’s law is almost equal to 1, thanks to the independence of the reads in the metagenomic data. However, in practice there is a slight overhead associated with the preprocessing involved in splitting the data (that is, deciding which worker nodes does what) and transferring the data to the nodes. With regard to the expectations from Amdahl’s law on the optimization of the three major contributors to the total run time of Tentacle, it is evident that reducing the time consumption of any of the three major run time contributors by parallelization would bring improvements to how Tentacle’s total run time is reduced. Currently, coverage computation is the foremost candidate for this parallelization.

One alternative to Tentacle is the MG-RAST (Meta Genome Rapid Annotation using Subsystem Technology) metagenomics analysis pipeline [49]. MG-RAST is a web-based service where users can upload their samples to the MG-RAST servers for analysis. MG-RAST offers a wide range of tools, including sequence quality assessment, read alignment, and gene quantification. The MG-RAST pipeline initially used BLAST for sequence alignment but it has switched to BLAT [30] and Bowtie [50] in recent versions [49]. MG-RAST is, however, limited by its web-based interface. This requires users to upload their data to the MG-RAST servers, which can be problematic and slow for large datasets. In contrast, Tentacle offers a flexible framework for gene quantification in metagenomes that can be run on local computer hardware. In addition, Tentacle is modular and can use more recent mapping algorithms (for example GEM [32], RazerS 3 [33], and USEARCH [34]), which offer substantial improvements in sensitivity and speed compared to BLAST and BLAT. Another drawback of an online web-based interface, such as MG-RAST’s, is that the web-based interface (while extensive) does not provide the same level of flexibility as having a locally operated software that allows for extension or modification to fit specific requirements, which might not be possible to fit into a web-based interface.

Alignment and quantification efficiency are important aspects to take into consideration when using an automated analysis pipeline. Although Tentacle does not implement any new mapping algorithms, it does offer a wide selection of third party mappers that can be used. Therefore, the performance and accuracy of the mapping process depends entirely on what specific mapping software is chosen. Tentacle has modules for six different mapping programs: Bowtie 2 [29], GEM [32], pBLAT [31], RazerS 3 [33], USEARCH [34], and NCBI BLAST [14]. Although none of these tools are capable of running on a computer cluster out-of-the-box, some are parallelized so that they can run several concurrent threads to take advantage of several CPUs in a single computer. Each algorithm has inherent benefits and drawbacks, so the choice of mapping algorithm depends on the specific metagenomes analyzed and the hypotheses addressed [45]. Because the accuracy of alignment (and thus the gene quantification) depends heavily on the input data, the alignment algorithm chosen, and the alignment parameters used, the mapping results can vary substantially from case to case. For example, when short reads are mapped to *de novo* assembled contigs or reference genomes, few mismatches are typically expected and high specificity is more important. Accepting a decrease in sensitivity (which often also improves alignment speed) can, therefore, be satisfactory. However, when comparing longer sequence reads to more distantly related genes and genomes, a higher sensitivity will be necessary [46].

It is also important to note that it is often common to see ambiguously mapped reads with modern short read technology. This can complicate the analysis and make the results harder to interpret, especially for downstream analysis. The implementation in Tentacle allows users to select to either use the ‘best hit’ (as defined by each mapper) or use all hits that comply with matching criteria. This is the same approach that the widely used MG-RAST platform uses. The ability to choose essentially any mapper in Tentacle also contributes to making it suited for gene quantification of data generated from most metagenomic experiments, allowing users to pick an algorithm that best deals with the particular characteristics of their data.

Besides enabling high-throughput high-sensitivity read mapping of large metagenomic datasets, versatility and modularity were also two important objectives in the design of the Tentacle framework. To achieve this, calls to external software (such as mappers, sequence filters and job-scheduling tools) are placed in separate Python modules, making it easy to add, adapt, and implement support for other components if desired (such as to change quality assessment algorithms, mappers, or job-schedulers). In addition, the post-processing step that computes coverage is also modular, making it straight-forward to implement custom algorithms; for example, for coverage/binning calculations or to modify the computation of coverage statistics. Thus, Tentacle can with little effort be adapted to various applications of metagenomics and integrated with existing frameworks. It is also possible to extend the method to other situations where read alignment is computationally limiting when run on single-computer systems, such as genome resequencing or RNA sequencing. The Tentacle Python package is published under the GNU General Public License (version 3) and the code is publicly available in an online repository [51]. Consequently, researchers who use Tentacle and create or modify modules can contribute improvements back to the repository for the benefit of the research community.

In conclusion, Tentacle provides researchers with a novel framework to efficiently analyze and study large metagenomes. Tentacle’s flexibility and modularity makes it a versatile framework that can easily be adapted and integrated into existing workflows and data analysis pipelines. It runs on UNIX-based computers and computer clusters, and it scales very well with increasing computing resources, making it a cost-effective way to quantify genes in large metagenomic datasets.

## Availability and requirements

- **Project name:** Tentacle
- **Project home page:**http://bioinformatics.math.chalmers.se/tentacle
- **Operating system(s):** Linux, OS X
- **Programming language:** Python 2.7
- **Other requirements:** a supported sequence aligner (see Implementation).
- **License:** GNU GPL v3

## Availability of supporting data

An archived snapshot of the code and supporting materials used in this paper is available from the *GigaScience* GigaDB database [52].

## Additional files

Additional file 1 **Example of Tentacle quantification output.** (PDF 48.8 Kb)

Additional file 2 **Default mapper settings in Tentacle.** (PDF 74.5 Kb)

Additional file 3 **Comparison between Tentacle and CloVR.** (PDF 86 Kb)

Additional file 4 **Evaluation of counts/coverage accuracy.** (PDF 156 Kb)

## References

[1] Baker M. Next-generation sequencing: adjusting to data overload. Nature Methods. 2010;7. Available from: http://dx.doi.org/10.1038/nmeth0710-495.

[2] Cochrane G, Alako B, Amid C, Bower L, Ana C, Cleland I (2013). Facing growth in the European Nucleotide Archive. Nucleic Acids Res.

[3] Scholz M, Lo C, Chain P (2012). Next generation sequencing and bioinformatic bottlenecks: the current state of metagenomic data analysis. Curr Opin Biotechnol.

[4] Handelsman J (2004). Metagenomics: application of genomics to uncultured microorganisms. Microbiol Mol Biol Rev: MMBR.

[5] Gilbert J, Dupont C (2011). Microbial metagenomics: beyond the genome. Ann Rev Mar Sci.

[6] Qin J, Li R, Raes J, Arumugam M, Burgdorf K, Manichanh C (2010). A human gut microbial gene catalogue established by metagenomic sequencing. Nature.

[7] Turnbaugh P, Ley R, Hamady M, Claire F, Knight R, Gordon J (2007). The human microbiome project. Nature.

[8] Gilbert JA, Bailey M, Field D, Fierer N, Fuhrman JA, Hu B (2011). The Earth Microbiome Project: The meeting report for the 1st International Earth Microbiome Project Conference, Shenzhen, China, June 13th-15th 2011. Stand Genomic Sci.

[9] Gilbert J, Jansson J, Knight R (2014). The Earth Microbiome project: successes and aspirations. BMC Biology.

[10] Reddy TBK, Thomas AD, Stamatis D, Bertsch J, Isbandi M, Jansson J, et al. The Genomes OnLine Database (GOLD) v.5: a metadata management system based on a four level (meta)genome project classification. Nucleic Acids Res. 2014. Available from: http://dx.doi.org/10.1093/nar/gku950. 10.1093/nar/gku950 PMC4384021 25348402

[11] Curtis T, Sloan W, Scannell J (2002). Estimating prokaryotic diversity and its limits. Proc Natl Acad Sci USAs.

[12] Li H, Homer N (2010). A survey of sequence alignment algorithms for next-generation sequencing. Brief Bioinform.

[13] Hatem A, Bozdag D, Toland A, Çatalyürek UV (2013). Benchmarking short sequence mapping tools. BMC Bioinforma.

[14] Altschul S, Madden T, Schäffer A, Zhang J, Zhang Z, Miller W (1997). Gapped BLAST and PSI-BLAST: a new generation of protein database search programs. Nucleic Acids Res.

[15] Grant J, Dunbrack R, Manion F, Ochs M (2002). BeoBLAST: distributed BLAST and PSI-BLAST on a Beowulf cluster. Bioinformatics (Oxford, England).

[16] Carvalho P, Glória R, de Miranda A, Degrave W (2005). Squid - a simple bioinformatics grid. BMC bioinforma.

[17] Yang C, Han T, Kan H (2009). G-BLAST: a Grid-based solution for mpiBLAST on computational Grids. Concurr Comput: Pract Exper.

[18] Darling A, Carey L, Feng Wc. The design, implementation, and evaluation of mpiBLAST (Best Paper: Applications Track). 4th International Conference on Linux Clusters: The HPC Revolution 2003 in conjunction with ClusterWorld Conference & Expo. 2003:14.

[19] Wang J, Mu Q (2003). Soap-HT-BLAST: high throughput BLAST based on Web services. Bioinformatics (Oxford, England).

[20] Dowd S, Zaragoza J, Rodriguez J, Oliver M, Payton P (2005). Windows.NET network distributed basic local alignment search toolkit (W.ND-BLAST). BMC bioinformatics.

[21] Angiuoli SV, Matalka M, Gussman A, Galens K, Vangala M, Riley DR (2011). CloVR: a virtual machine for automated and portable sequence analysis from the desktop using cloud computing. BMC bioinformatics.

[22] Pandey RV, Schlötterer C (2013). DistMap: A toolkit for distributed short read mapping on a hadoop cluster. PLoS ONE.

[23] Schatz M (2009). CloudBurst: highly sensitive read mapping with MapReduce. Bioinformatics (Oxford, England).

[24] Nguyen T, Shi W, Ruden D (2011). CloudAligner: A fast and full-featured MapReduce based tool for sequence mapping. BMC research notes.

[25] Karczewski KJ, Fernald GH, Martin AR, Snyder M, Tatonetti NP, Dudley JT (2014). STORMSeq: an open-source, user-friendly pipeline for processing personal genomics data in the cloud. PLoS ONE.

[26] Caporaso JG, Kuczynski J, Stombaugh J, Bittinger K, Bushman FD, Costello EK (2010). QIIME allows analysis of high-throughput community sequencing data. Nat Methods.

[27] iMatix Corporation. ZeroMQ. 2014. Available from: http://www.zeromq.org/. Accessed 22 Aug 2015.

[28] Hannon lab. FASTX-Toolkit. 2014. Available from: http://hannonlab.cshl.edu/fastx_toolkit. Accessed 22 Aug 2015.

[29] Langmead B, Salzberg S (2012). Fast gapped-read alignment with Bowtie 2. Nat methods.

[30] Kent W (2002). BLAT–the BLAST-like alignment tool. Genome res.

[31] Meng W. pblat – blat with multi-threads support. 2015. Available from: http://icebert.github.io/pblat/. Accessed 22 Aug 2015.

[32] Santiago M, Sammeth M, Guigó R, Ribeca P (2012). The GEM mapper: fast, accurate and versatile alignment by filtration. Nat methods.

[33] Weese D, Holtgrewe M, Reinert K (2012). RazerS 3 faster, fully sensitive read mapping. Bioinformatics (Oxford, England).

[34] Edgar RC (2010). Search and clustering orders of magnitude faster than BLAST. Bioinformatics.

[35] Boulund F, Sjögren A, Kristiansson E. Tentacle. 2014. Available from: http://bioinformatics.math.chalmers.se/tentacle/. Accessed 22 Aug 2015. 10.1186/s13742-015-0078-1 PMC4562114 26351566

[36] SchedMD. Slurm. 2014. Available from: http://slurm.schedmd.com/. Accessed 22 Aug 2015.

[37] Pérez F, Granger BE (2007). IPython: a System for Interactive Scientific Computing. Comput Sci Eng.

[38] Boulund F, Sjögren A, Kristiansson E. Tentacle scaling benchmark. 2015. Available from: http://dx.doi.org/10.6084/m9.figshare.1403608. 10.1186/s13742-015-0078-1 PMC4562114 26351566

[39] Atallah MJ. Algorithms and theory of computation handbook: Danvers, MA: CRC press; 1998.

[40] Forum MPI. MPI: A message-passing interface standard. Version 3.0. 2012. Available from: http://www.mpi-forum.org/docs/mpi-3.0/mpi30-report.pdf.

[41] Gottumukkala N, Nassar R, Paun M, Leangsuksun C, Scott S (2010). Reliability of a System of k Nodes for High Performance Computing Applications. IEEE Trans Reliab.

[42] Armbrust M, Fox A, Griffith R, Joseph AD, Katz R, Konwinski A (2010). A view of cloud computing. Commun ACM.

[43] Dean J, Ghemawat S (2008). MapReduce: simplified data processing on large clusters. Commun ACM.

[44] White T (2012). Hadoop: The definitive guide.

[45] Mande S, Mohammed M, Ghosh T (2012). Classification of metagenomic sequences: methods and challenges. Brief Bioinform.

[46] Schbath S, Martin V, Zytnicki M, Fayolle J, Loux V, Gibrat J (2012). Mapping reads on a genomic sequence: an algorithmic overview and a practical comparative analysis. J Comput Biol: J Mol Cell Biol.

[47] Roguski L, Deorowicz S (2014). DSRC 2–Industry-oriented compression of FASTQ files. Bioinformatics.

[48] Rodgers DP (1985). Improvements in Multiprocessor System Design. SIGARCH Comput Archit News.

[49] Meyer F, Paarmann D, D’Souza M, Olson R, Glass E, Kubal M (2008). The metagenomics RAST server - a public resource for the automatic phylogenetic and functional analysis of metagenomes. BMC Bioinformatics.

[50] Langmead B, Trapnell C, Pop M, Salzberg S (2009). Ultrafast and memory-efficient alignment of short DNA sequences to the human genome. Genome Biol.

[51] Boulund F, Sjögren A, Kristiansson E. Tentacle open source repository at Bitbucket. 2014. Available from: http://www.bitbucket.org/chalmersmathbioinformatics/tentacle. Accessed 22 Aug 2015.

[52] Boulund F, Sjögren A, Kristiansson E. Supporting materials and software for “Tentacle: distributed quantification of genes in metagenomes”. 2015. GigaScience Database. http://dx.doi.org/10.5524/100152. 10.1186/s13742-015-0078-1 PMC4562114 26351566

[53] Kristiansson E. 1928 Diagnostics. Resqu. 2014. Available from: http://www.1928diagnostics.com/resdb/. Accessed 22 Aug 2015.

