# Supplementary material for: Tentacle: distributed quantification of genes in metagenomes
Source: Gigascience. 2015 Sep 7;4:40. doi: 10.1186/s13742-015-0078-1 (PMC4562114; doi:10.1186/s13742-015-0078-1)
Supplement: Additional file 1 — Example of Tentacle quantification output. (PDF 48.8 Kb) [file 13742_2015_78_MOESM1_ESM.pdf]

Additional file 1 — Example of Tentacle quantification output

An example of Tentacle's quantification output. A plain text file with five columns (one description per line):

```
reference_annotation:start:stop:strand
counts
median coverage
average coverage
coverage standard deviation
```

In the following output, contigs 4 and 5 have been spiked with randomly drawn reads, which are drawn from the same contigs to produce an additional 5x coverage of these two contigs.

```
contig3_c3:0:562:+     8      1.0     1.08185053381    1.4491590858
contig2_c2:0:632:+     9      1.0     1.08227848101    0.785269363391
contig1_c1:0:623:+     11     1.0     1.341894061      1.13613956271
contig5_c5:0:2015:+    150    6.0     5.65756823821    2.5727838843
contig4_c4:0:1884:+    155    6.0     6.25265392781    2.81183961158
```
