# Supplementary material for: Tentacle: distributed quantification of genes in metagenomes
Source: Gigascience. 2015 Sep 7;4:40. doi: 10.1186/s13742-015-0078-1 (PMC4562114; doi:10.1186/s13742-015-0078-1)
Supplement: Additional file 2 — Default mapper settings in Tentacle. (PDF 74.5 Kb) [file 13742_2015_78_MOESM2_ESM.pdf]

Additional file 2 — Default mapper settings in Tentacle

The following subsections list the default settings for each mapper in their respective Tentacle modules. The equivalent command lines are shown below. Note, however, that all of the settings can be completely customized by the user and custom mapping modules can be created that implement any combination of pre- or post-processing of the data, as well as including arguments to the program calls that are not included in the listing below. All of the default settings listed below are for Tentacle version 0.1.0b.

Bowtie2

The default settings for bowtie2 makes a search for multiple alignments of each read but reports only the best. Bowtie 2 version 2.1.0 was used during the development and evaluation of Tentacle.

`bowtie2 -x DBNAME -S OUTPUT -U INPUT -p N`,

where DBNAME, OUTPUT, INPUT are the filenames of the reference database, output file, input file, respectively. N is the number of processors available on the current computer/node (automatically determined with psutil).

GEM

The default settings for GEM search for the best alignment of each read with at least 80% matches bases and maximum 4% mismatches (with maximum edit distance 4%). GEM version 1.376 beta was used during the development and evaluation of Tentacle.

`gem-mapper -I DBNAME -i INPUT -o OUTPUT -T N -m M -e E --min-matched-bases B --granularity G`,

where DBNAME, INPUT, OUTPUT are the filenames of the reference database, input file, output file, respectively. N is the number of processors available on the current computer/node (automatically determined with psutil). M is the maximum number of mismatches and is default 0.04, E is the maximum edit number and is default 0.04, B is the minimum number of match bases and defaults to 0.80, G is the granularity when reading from the file and defaults to 2,500,000 bytes. If the input files are in the FASTA format, then the following flag is added: `-q ignore`.

pBLAT

The default settings for pBLAT search for the best match of each read with a minimum identity of 90%. pBLAT version v.34 and v.35 were used during the development and evaluation of Tentacle.

`pblat -threads=N -minIdentity=I -out=blast8`,

where N and I are the number of threads and the minimum sequence identity, respectively. The default number of threads is automatically determined using psutil as the number of processors available on the current computer/node. The minimum identity is default 90%.

RazerS 3

The default settings for RazerS 3 makes a search for the best match for each read, with a minimum identity of 95%. RazerS 3 version 3.2 was used during the development and evaluation of Tentacle.

`razers3 -i I -rr R -m M -tc N DBNAME INPUT OUTPUT`,
where DBNAME, INPUT, OUTPUT are the filenames of the reference database, input file, and output file, respectively. I is the percent identity of matches reads and defaults to 95, R is the recognition rate and defaults to 100, M is the maximum number of returned matches for each read and defaults to 1, and N is the number of processors available on the current computer/node (automatically determined with psutil).

USEARCH
The default settings for USEARCH are set to search against a protein database with both strands of the translated reads. The entire read must be aligned (coverage 100%) with an identity of 90%. USEARCH version v7.0.1001 was used during the development and evaluation of Tentacle.

`usearch -usearch_local INPUT -query_cov C -db DBNAME -id I -blast6out OUTPUT -strand S`,
where INPUT, DBNAME, OUTPUT are the filenames of the input file, reference database, output file, respectively. C is the query coverage criterion and defaults to 1.0. I is the identity criterion and defaults to 0.9, S is which strand to search and defaults to 'both'.

NCBI BLAST
The default settings for BLAST makes a search with BLASTN using megablast. Version 2.2.28+ was used during the development and evaluation of Tentacle.

`blastn -outfmt F -query INPUT -db DBNAME -out OUTPUT -num_threads N`,
where INPUT, DBNAME, OUTPUT are the filenames of the input file, reference database, output file, respectively. F is the output format and defaults to 6 (BLAST tabular output), N is the number of processors available on the current computer/node (which is automatically determined with psutil).
