# Supplementary material for: Tentacle: distributed quantification of genes in metagenomes
Source: Gigascience. 2015 Sep 7;4:40. doi: 10.1186/s13742-015-0078-1 (PMC4562114; doi:10.1186/s13742-015-0078-1)
Supplement: Additional file 3 — Comparison between Tentacle and CloVR. (PDF 86 Kb) [file 13742_2015_78_MOESM3_ESM.pdf]

Additional file 3 — Comparison between Tentacle and CloVR

Test design

CloVR is only capable of running BLAST. The performance of CloVR running BLAST was compared to the performance of Tentacle running BLAST on the same data. For comparison purposes, the run times with BLAST are also contrasted with the run time of Tentacle using pBLAT.

The test machine was a 32 core Red Hat Enterprise Linux Server (6.6) with 192 GiB of RAM. CloVR (version `clovr-standard-2014-10-07-21-11-54`) was run using Oracle VirtualBox (version `4.3.20 r96996`) with an allocation of 16 CPUs and 131072 MiB of memory, all of the other settings were as recommended by the CloVR documentation. CloVR uses `blastall` (version `2.2.21`). Tentacle was run using BLAST (`blastn` version `2.2.30+`) and pBLAT (version `v. 35`) [1].

CloVR automatically splits the input files into evenly-sized chunks, in this case 2.7 MiB size parts (25134 reads each). To make the comparison as fair as possible, Tentacle's input file was manually split into 466 parts, just as CloVR does. When running Tentacle with pBLAT, no file splitting was performed because this is not required when using pBLAT.

Test data

The test data was a single read file, MH0050_081223.raw.1.fq [2], and the contigs were assembled from that sample (this specific sample consists of four files in total). The reads file contains 11680508 reads that are all 45 bases long. The contigs file contains 71,587 contigs with an average length of 1523 base pairs.

Results

Table 1 Tentacle vs CloVR. All of the use cases used the same read data, consisting of 11,680,508 reads with a total size of 1.3 GiB in FASTA format [2]. The examples were run on the same machine and with the same amount of computing resources. The default BLAST options were used when running BLAST in CloVR: `-p blastn -m8 -e 1e-5`. The following options were used in Tentacle, BLAST: `-outfmt 6 -num_threads 16 -task blastn`; pBLAT: `-threads=16 -minIdentity=90 -out=blast8`

Framework	Mapper	Run time (wall-clock)
CloVR	BLAST	15h 9m 8s
Tentacle	BLAST	5h 7m 0s
Tentacle	pBLAT	0h 6m 43s

Author details

References

1. Fredrik Boulund, Anders Sjögren, and Erik Kristiansson. Supporting materials and software for "tentacle: distributed quantification of genes in metagenomes", jul 2015.
2. Junjie Qin, Ruiqiang Li, Jeroen Raes, Manimozhiyan Arumugam, Kristoffer Burgdorf, Chaysavanh Manichanh, Trine Nielsen, Nicolas Pons, Florence Levenez, Takuji Yamada, Daniel Mende, Junhua Li, Junming Xu, Shaochuan Li, Dongfang Li, Jianjun Cao, Bo Wang, Huiqing Liang, Huisong Zheng, Yinlong Xie, Julien Tap, Patricia Lepage, Marcelo Bertalan, Jean-Michel Batto, Torben Hansen, Denis Le Paslier, Allan Linneberg, H Nielsen, Eric Pelletier, Pierre Renault, Sicheritz-Ponten, Thomas, Keith Turner, Hongmei Zhu, Chang Yu, Shengting Li, Min Jian, Yan Zhou, Yingrui Li, Xiuqing Zhang, Songgang Li, Nan Qin, Huanming Yang, Jian Wang, Søren Brunak, Joel Doré, Francisco Guarner, Karsten Kristiansen, Oluf Pedersen, Julian Parkhill, Jean Weissenbach, MetaHIT Consortium, Peer Bork, S Ehrlich, and Jun Wang. A human gut microbial gene catalogue established by metagenomic sequencing. *Nature*, 464(7285):59–65, 2010.
