# Supplementary material for: Tentacle: distributed quantification of genes in metagenomes
Source: Gigascience. 2015 Sep 7;4:40. doi: 10.1186/s13742-015-0078-1 (PMC4562114; doi:10.1186/s13742-015-0078-1)
Supplement: Additional file 4 — Evaluation of counts/coverage accuracy. (PDF 156 Kb) [file 13742_2015_78_MOESM4_ESM.pdf]

Additional file 4 — Evaluation of counts/coverage accuracy

Three samples were randomly chosen from Qin et al. (2010) [3]. From these three samples, 10% of contigs longer than 2,500 nucleotides were randomly chosen for spiking. The contigs selected for spiking were spiked with synthetic reads that were randomly drawn from the same contigs. The contigs were spiked to 1X, 10X, 100X, and 1,000X coverage. The spiked samples were mapped back to the contigs using pBLAT with Tentacle using default settings. The results were normalized by subtracting the counts/coverage values measured in the non-spiked samples. Complete details of how the evaluation was performed, along with calculations, are available in an IPython notebook [1] [http://dx.doi.org/10.6084/m9.figshare.1403609].

Author details

References

1. Fredrik Boulund, Anders Sjögren, and Erik Kristiansson. Supporting materials and software for "tentacle: distributed quantification of genes in metagenomes", jul 2015.
2. Jerry L Hintze and Ray D Nelson. Violin plots: a box plot-density trace synergism. *The American Statistician*, 52(2):181–184, 1998.
3. Junjie Qin, Ruiqiang Li, Jeroen Raes, Manimozhiyan Arumugam, Kristoffer Burgdorf, Chaysavanh Manichanh, Trine Nielsen, Nicolas Pons, Florence Levenez, Takuji Yamada, Daniel Mende, Junhua Li, Junming Xu, Shaochuan Li, Dongfang Li, Jianjun Cao, Bo Wang, Huiqing Liang, Huisong Zheng, Yinlong Xie, Julien Tap, Patricia Lepage, Marcelo Bertalan, Jean-Michel Batto, Torben Hansen, Denis Le Paslier, Allan Linneberg, H Nielsen, Eric Pelletier, Pierre Renault, Sicheritz-Ponten, Thomas, Keith Turner, Hongmei Zhu, Chang Yu, Shengting Li, Min Jian, Yan Zhou, Yingrui Li, Xiuqing Zhang, Songgang Li, Nan Qin, Huanming Yang, Jian Wang, Søren Brunak, Joel Doré, Francisco Guarner, Karsten Kristiansen, Oluf Pedersen, Julian Parkhill, Jean Weissenbach, MetaHIT Consortium, Peer Bork, S Ehrlich, and Jun Wang. A human gut microbial gene catalogue established by metagenomic sequencing. *Nature*, 464(7285):59–65, 2010.

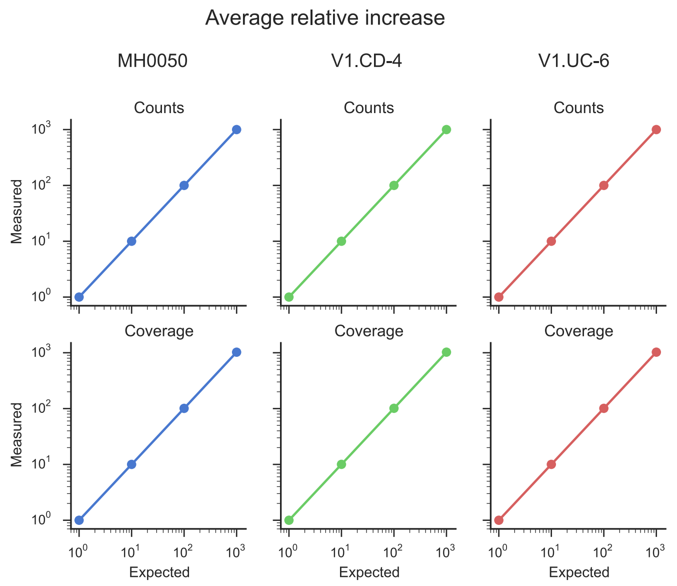

Figure 1 Counts/coverage accuracy evaluation. Measured versus expected counts/coverage for each sample. For complete details, see the IPython notebook.

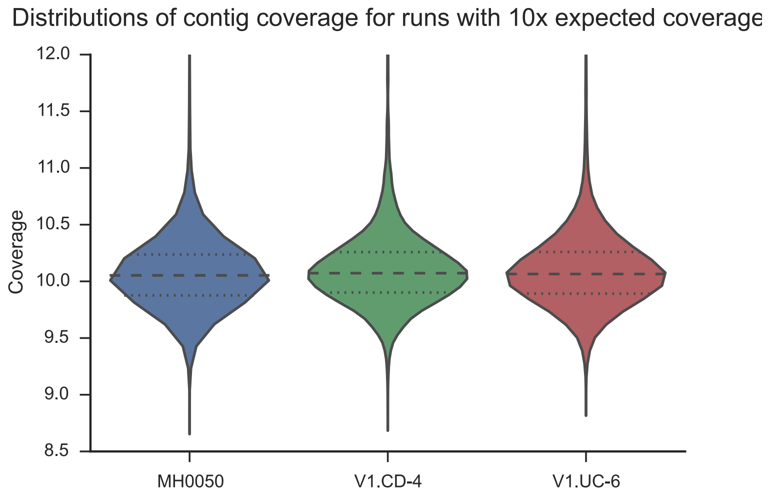

Figure 2 Distributions of contig coverage for runs with 10X expected coverage. Violin plots [2] of coverage distribution for contigs with 10X expected coverage after normalization. Note that the averages are very close to 10X. Some outliers with values above coverage 12X are not displayed. MH0050 : 51 outlier points above coverage 12 (7169 points total), V1.CD-4: 22 outlier points above coverage 12 (6485 points total), V1.UC-6: 28 outlier points above coverage 12 (8865 points total). For complete details, please see the IPython notebook.
